# Molecular Insights into the Classification of Luminal Breast Cancers: The Genomic Heterogeneity of Progesterone-Negative Tumors

**DOI:** 10.3390/ijms20030510

**Published:** 2019-01-25

**Authors:** Gianluca Lopez, Jole Costanza, Matteo Colleoni, Laura Fontana, Stefano Ferrero, Monica Miozzo, Nicola Fusco

**Affiliations:** 1 Division of Pathology, Fondazione IRCCS Ca’ Granda-Ospedale Maggiore Policlinico, 20122 Milan, Italy; gianluca.lopez@unimi.it (G.L.); matteo.colleoni1@studenti.unimi.it (M.C.); stefano.ferrero@unimi.it (S.F.); 2 School of Pathology, University of Milan, 20122 Milan, Italy; 3 Research Laboratory Unit, Fondazione IRCCS Ca’ Granda Ospedale Maggiore Policlinico, 20122 Milan, Italy; jole.costanza@policlinico.mi.it (J.C.); monica.miozzo@unimi.it (M.M.); 4 Medical Genetics, Department of Pathophysiology and Transplantation, University of Milan, 20122 Milan, Italy; laura.fontana@unimi.it; 5 Pathology, Department of Biomedical, Surgical, and Dental Sciences, University of Milan, 20122 Milan, Italy

**Keywords:** breast cancer, progesterone receptor negative, mutational profiling, PI3K pathway, TP53

## Abstract

Estrogen receptor (ER)-positive progesterone receptor (PR)-negative breast cancers are infrequent but clinically challenging. Despite the volume of genomic data available on these tumors, their biology remains poorly understood. Here, we aimed to identify clinically relevant subclasses of ER+/PR− breast cancers based on their mutational landscape. The Cancer Genomics Data Server was interrogated for mutational and clinical data of all ER+ breast cancers with information on PR status from The Cancer Genome Atlas (TCGA), Memorial Sloan Kettering (MSK), and Molecular Taxonomy of Breast Cancer International Consortium (METABRIC) projects. Clustering analysis was performed using gplots, ggplot2, and ComplexHeatmap packages. Comparisons between groups were performed using the Student’s *t*-test and the test of Equal or Given Proportions. Survival curves were built according to the Kaplan–Meier method; differences in survival were assessed with the log-rank test. A total of 3570 ER+ breast cancers (PR− *n* = 959, 27%; PR+ *n* = 2611, 73%) were analyzed. Mutations in well-known cancer genes such as *TP53*, *GATA3*, *CDH1*, *HER2*, *CDH1*, and *BRAF* were private to or enriched for in PR− tumors. Mutual exclusivity analysis revealed the presence of four molecular clusters with significantly different prognosis on the basis of *PIK3CA* and *TP53* status. ER+/PR− breast cancers are genetically heterogeneous and encompass a variety of distinct entities in terms of prognostic and predictive information.

## 1. Introduction

Estrogen receptor (ER)-positive progesterone receptor (PR)-negative (ER+/PR−) breast cancers are a subset of Luminal B tumors characterized by the strong and diffuse nuclear expression of ER-alpha but not of PR [1]. They account for 5% of all invasive breast cancers and show a relatively aggressive clinical course compared to ER+/PR+ neoplasms [1,2,3,4,5]. ER+/PR− invasive breast cancers are described as larger in size than PR+ carcinomas and are generally of no special histological type (i.e., ductal) [1,6]. Even though they preferentially affect postmenopausal women, these diagnoses are not exceptional in younger patients [1,2,7]. As confirmed by several prospectively randomized controlled neoadjuvant trials, ER+/PR− breast cancers are associated with a higher response but also worse long-term outcome after neoadjuvant therapy [5]. There are several lines of evidence to suggest that the worse prognosis of ER+/PR− tumors may be related to the phenomena of hormone therapy resistance [1,2,3,4,5]. However, a large adjuvant trial on the use of aromatase inhibitors in postmenopausal women with early breast cancer revealed that the PR status has no effect on the relative efficacy of this therapy [8]. For this reason, some authors have questioned the clinical utility of PR testing [9]. To date, hormonal therapy remains recommended in ER+ tumors regardless of PR status [10]. All these diverse correlations highlight the clinical challenges provided by ER+/PR− breast cancers.

A proportion of ER+/PR− neoplasms shows a remarkable degree of genomic instability, reaching almost twice the DNA copy number variations and tumor mutational load than those of both ER+/PR+ and ER− breast cancers [1,8]. Furthermore, many growth factors were observed to be overexpressed in these tumors, such as HER family, PI3K, Akt, and src [1,2,11,12,13]. These pathways, which can also be altered in ER+/PR+ tumors, are known to be involved in ER phosphorylation, which may lead to ligand-independent activation [14]. There is also evidence that the upregulation of Akt and HER1/2 is implicated in tamoxifen resistance [1,2,11,12,15,16,17,18]. Recently, PR has been proposed as a surrogate biomarker of altered growth factor signaling [5]. Due to these insights, and the substantial lack of distinct biological properties identified to date in ER+/PR− breast cancers, it is becoming increasingly clear that these tumors are clinically and biologically heterogeneous [19,20,21,22,23,24,25]. 

During the past few years, the Cancer Genome Atlas (TCGA) project has exposed the complexity of the genome-wide genetic alterations in breast cancer [26]. On the other hand, the proper clinical management of Luminal (i.e., ER+) breast cancers, particularly in intermediate-risk patients, remains a matter of controversy. However, there is a limited understanding of how the mutational landscape of these tumors, according to the PR status, can be exploited in the clinic to allow for more tailored management schemes. In this study, we sought: (i) to characterize the mutational signatures of ER+/PR− breast cancers; (ii) to compare the molecular landscapes of PR− and PR+ Luminal tumors; and (iii) to define the prognostic value of the type and pattern of somatic genetic alterations in these patients.

## 2. Results

A total of 3589 ER+ breast cancers from the publicly available datasets TCGA, Memorial Sloan Kettering (MSK), and Molecular Taxonomy of Breast Cancer International Consortium (METABRIC) were identified. Among them, 3570 (99.5%) cases (2815 invasive ductal carcinomas and 755 invasive carcinomas of any special type) had information on PR status (PR− *n* = 959, 27%; PR+ *n* = 2611, 73%) and were included in the current study. The median age at diagnosis of PR− tumors was 59 years old (range 24–92); for PR+ tumors, it was 57 years old (range 23–91). Taken together, 53,585 mutations targeting 13,402 genes were identified, including 57,448 (99%), 6642 (90%), and 8905 (89%) mutations that were private to only one sample in the TCGA, MSK, and METABRIC cohorts, respectively. The number of samples, mutated genes, and mutations of the tumors included in the analysis are summarized in Table 1 and Table S1. 

### 2.1. The Molecular Landscape of ER+/PR− Breast Cancers

**Figure 1:**
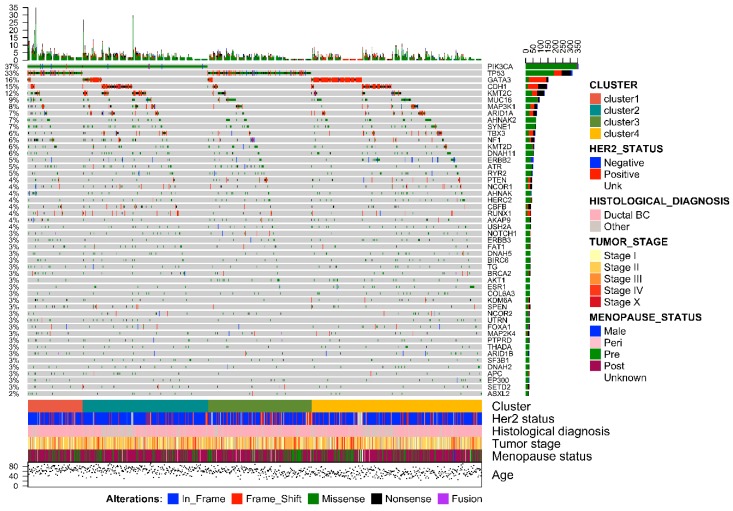
Oncoprint visualization of highly recurrent somatic molecular alterations in ER+/PR− breast cancers (959 samples). Each row represents a gene, as reported on the right, and was sorted by gene alterations frequency (bar plot on the right); types of alterations are color-coded on the basis of the legend on the bottom. Each column represents a sample and was sorted to appreciate the mutual exclusivity across genes. The bar plot on the top represents the number of samples showing alterations in the displayed genes. Cluster analysis, human epidermal growth factor receptor (HER)2 status, histological type, tumor stage, menopause status, and age at diagnosis are reported as rows at the bottom of the figure. Clustering was performed according to the mutual exclusivity and patterns of mutations.

The average number of mutations displayed by ER+/PR− breast cancers was 16 per sample, whereas in PR+ tumors was 14. The two groups shared 5668 mutated genes, while approximately 1319 (19%) genes were found to be privately altered in ER+/PR− breast cancers. Overall, the mutations in PR− tumors were missense in 12,583 (78%), nonsense in 1250 (8%), frameshift deletions in 896 (5%), frameshift insertions in 616 (4%), splicing in 516 (3%), and in-frame indels in 261 (2%) cases. Of note, fusion genes were detected in 69 ER+/PR− tumors. The mutational landscape and selected clinicopathologic features in ER+/PR− and ER+/PR+ breast cancers are depicted in Figure 1 and Figure S1, respectively. 

**Figure 2:**
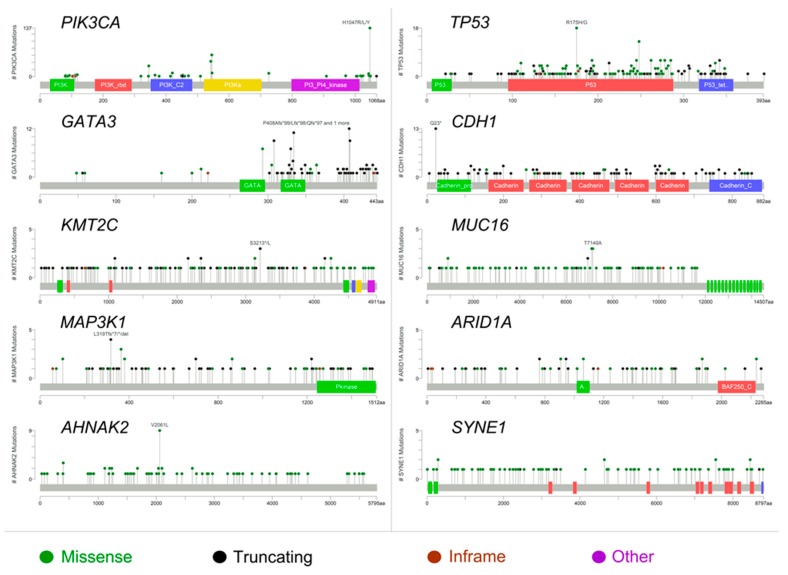
Type of mutations and affected protein domains of the 10 most frequently altered genes in ER+/PR− breast cancers. Mutation types are color-coded on the basis of the legend at the bottom.

The most frequently mutated gene in PR− tumors was phosphatidylinositol-4,5-bisphosphate 3-kinase, catalytic subunit alpha (*PIK3CA*), with lower prevalence than in PR+ tumors (*n* = 354, 37% vs. *n* = 1220, 47%; *p* < 0.01). In particular, the vast majority of *PIK3CA* mutations were missense and affected four hotspot regions of the gene, namely N345K, E542K, E545K, and H1047R (Figure 2). Notably, the H1047R and E545K mutations in *PIK3CA* were less frequent in PR− tumors (Table 2). The prevalence of samples showing mutations in *TP53*, which was the second most frequently mutated gene in both PR− and PR+ Luminal tumors, was higher in PR− breast cancers (*n* = 312, 33% vs. *n* = 496, 19%; *p* < 0.01). Furthermore, the nonsense mutation R342X and the missense mutations P728S, I195T, and H179R in *TP53* were enriched in PR− tumors (*p* < 0.05), as shown in Table 2. Taken together, *PIK3CA* and *TP53* status allowed for the definition of four molecular clusters (Figure 1). Specifically, Cluster 1 included all *PIK3CA*-mutant/*TP53*-mutant samples (*n* = 108, 11%), Cluster 2 all *PIK3CA*-mutant/*TP53* wild-type samples (*n* = 246, 26%), Cluster 3 *PIK3CA* wild-type/*TP53*-mutant tumors (*n* = 204, 21%), and Cluster 4 encompassed all *PIK3CA*/*TP53* wild-type cases (*n* = 401, 42%). Among the other recurrent gene alterations, the hotspot mutation E17K in RAC-alpha serine/threonine-protein kinase (*AKT1*), which was present in 3% and 5% of PR− and PR+ cases, respectively, was mutually exclusive with mutations targeting *PIK3CA*, regardless of PR status (Figure S2). On the other hand, even if *PIK3CA* and *AKT1* were observed to be recurrently mutated in both groups, the hotspot regions differed significantly on the basis of PR activation (*p* < 0.05). Of note, *GATA3* showed a high number of frame-shift indels and nonsense mutations (Figure 2), consistent with its crucial role in the ER signaling pathway. One of the most recurrently mutated genes was E-cadherin (*CDH1*), with the hotspot truncating mutation in position 23 associated to the lobular histology (Figure 2). The prevalence of human epidermal growth factor receptor (HER)2-mutant cases was higher in PR− breast cancers, albeit nonsignificant (*n* = 151, 16% vs. *n* = 389, 15%; *p* = 0.530). According to the Student’s *t*-test, the mutational profile of PR− Luminal breast cancers was significantly different to that of PR+ tumors (*p* < 10^−5^), with 16 mutations being restricted to the ER+/PR− group, including mutations in *ARID1A*, *ATR*, *BCL6*, *BRAF*, *CARD11*, *CDH1*, *AXIN2*, *GATA3*, *MUC16*, *CCDC82*, *RUNX1*, and *TBX3* (Table 2). No significant correlations were observed between PR activation status and other clinicopathologic characteristics. The tumor mutational burden (median of five mutations per sample for both PR+/−; mean 15.2 per sample for PR+; mean 15.9 per sample for PR−; range 1–3474 in PR+; and range 1–2900 in PR−) of the cases included in this study is shown in Figure S3.

### 2.2. The Prognostic Role of PIK3CA and TP53 in ER+/PR− Breast Cancers

**Figure 3:**
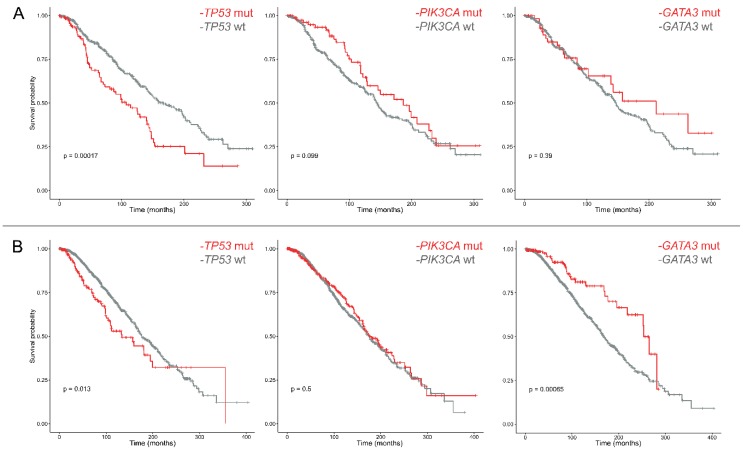
Overall survival of ER+/PR− (**A**) and ER+/PR+ (**B**) breast cancer patients based on *TP53*, *PIK3CA*, and *GATA3* gene alterations. For each analysis, all samples harboring mutations in one of the other two genes were excluded. Survival curves are built according to the Kaplan–Meier method.

Overall, the highest mortality was observed before 50 months from the diagnosis, regardless of PR status, with a median survival of 76.9 months in PR− and 61 months in PR+ tumors. The most recurrently mutated genes in ER+/PR− and ER+/PR+ breast cancers were used to define the survival probability curves shown in Figures S4 and S5, respectively. Even though the log-rank *p*-values were significant for *TP53* and *GATA3* mutations in both groups, survival analyses including tumors harboring alterations only in each of the most frequently mutated genes, but not in the others, revealed that in ER+/PR− breast cancers only *TP53* mutations are related to a different prognosis (Figure 3). The hotspot regions of *TP53* that were significantly different in PR− tumors were not related to a different outcome (Figure S6), similar to *PIK3CA* (Figure S7). 

**Figure 4:**
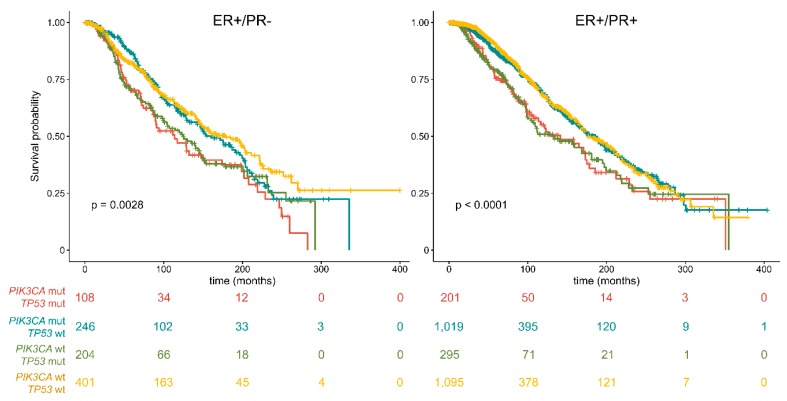
Overall survival of *PIK3CA*/*TP53*-mutant (Cluster 1), *PIK3CA*mutant/*TP53* wild-type (Cluster 2), *PIK3CA* wild-type/*TP53*-mutant (Cluster 3), and *PIK3CA*/*TP53* wild-type (Cluster 4) ER+ breast cancers, based on PR activation. Survival curves are built according to the Kaplan–Meier method.

Subsequently, survival curves were built according to the four molecular clusters identified on the basis of *PIK3CA* and *TP53* status. These analyses revealed the prognostic value of the combination and mutual exclusivity of *PIK3CA* and *TP53* mutations (Figure 4). Specifically, Cluster 4 showed in both PR− and PR+ cases a good prognosis. Interestingly, the prognosis of Cluster 4 overlapped to that of Cluster 3 in PR+ but not in PR− tumors. Hence, PR− breast cancers showed a different scenario, where the long-term outcome of the patients was worse in the presence of *PIK3CA* and/or *TP53* mutations (i.e., Clusters 1, 2 and 3).

## 3. Discussion

The precise risk stratification in Luminal breast cancer by means of immunohistochemistry and/or prognostic genomic tests is a major limitation in defining the most appropriate management scheme [27]. Patients with ER+ breast cancers are assumed to have a good prognosis, but the lack of PR expression may contribute to their poor outcomes. This may be a result of the de-differentiation of hormone-positive neoplasms and subsequent development of resistance phenomena to both anti-estrogen therapy and chemotherapy. Studies aiming to explore the genetic alterations in ER+/PR− breast cancers have been performed. However, the unique biology and challenging clinical course of these tumors, particularly in long-term survivors, suggest that they warrant further characterization. In this study, we analyzed a large cohort of PR− Luminal breast cancers with publicly available genomic data and compared their molecular landscape and prognosis to those of PR+ tumors. Altogether, we observed that several alterations in clinically actionable cancer genes are private to or enriched for in PR− breast cancers, such as *TP53* R342X, P728S, I195T, and H179R, *GATA3*, *CDH1*, *HER2*, *CDH1*, and *BRAF* V600E. Furthermore, we identified four molecular clusters on the basis of *PIK3CA* and *TP53* status with significantly different risk of death in PR− tumors.

Decreased expression and/or downregulation of PR in breast cancer leads to a subset of tumors that is phenotypically ER+/PR−. Even though several hypotheses to explain this phenomenon have been put forward, we are still far from fully understanding its biology. In a proportion of Luminal tumors, ER, although expressed, is biologically nonfunctional and therefore it is unable to stimulate PR production, particularly in postmenopausal women [1]. Another mechanism for PR loss is the epigenetic inactivation of its promoter through hypermethylation [12]. Even though a genetic loss of a PR gene locus has previously been observed [12], in our analysis, all ER+/PR− tumors are PR wild-type, suggesting that PR downregulation may be determined by growth factor pathways, as previously observed [2,11]. In particular, the HER2 activity may lead to the cytoplasmic sequestration of ER, which alters a set of genes that are normally regulated by ER, including PR−related genes, such as *PIK3CA* [11,28,29]. 

Taken together, we observed that the most frequently mutated genes in ER+/PR− breast cancers are *PIK3CA*, *TP53*, *GATA3*, *CHD1*, *KMT2C*, *MUC16*, *MAP3K1*, *ARID1A*, *AHNAK2*, and *SYNE2*. Interestingly, *PIK3CA* and *TP53* show a mutational prevalence (37% and 33%, respectively) that differs significantly to that of ER+/PR+ tumors (with *PIK3CA* mutated in 47% of cases and *TP53* in 19%). Those aspects have already been described in the literature [30,31]. On the other hand, the identification of a mutational profile specific to ER+/PR− cases, with 16 mutations being restricted to this group, is a novel finding. In our study, we confirm the presence of highly recurrent molecular alterations of the *PIK3CA* gene in position 1047, which likely constitute the driving genetic event in the pathogenesis of a subset of ER+/PR− breast cancers. These data provide further credence to the notion that inhibitors of this pathway (e.g., XL147) could reverse PR downregulation and overcome resistance to anti-HER2 drugs [32]. In addition, the identification of the *BRAF* V600E as a private mutation of PR− cases have possible therapeutic implications [33,34]. Recently, mutations in *HER2* have been detected in breast cancer patient samples which lack *HER2* gene amplification. Thirteen *HER2* mutations were characterized from twenty-five patient samples which had HER2 mutations but lacked HER2 gene amplification. Among them, seven mutations were activating and resulted from point mutations and in-frame deletions. Some mutations (L755S) resulted in lapatinib resistance; however, this was not an activating mutation. All of the cells containing the *HER2* mutations were sensitive to the irreversible HER2 kinase inhibitor, neratinib [35]. Our analysis corroborates the concept that mutations in *GATA3* are associated with a better outcome in ER+ breast cancer patients [36]. After eliminating all cases with concurrent mutations in the other top recurrently mutated genes, however, we were able to confirm this notion only in PR+ tumors. These data suggest that *GATA3* mutations are not an independent good prognostic factor in ER+/PR− tumors. Given that *GATA3* is frequently altered in Luminal A breast cancers, our findings provide an additional molecular layer to the worse prognosis showed by ER+/PR− breast cancers [19,37]. Furthermore, we confirmed that *TP53* mutations are associated with PR negativity and with a shorter overall survival time in breast cancers [38]. Interestingly, this behavior is unrelated to the specific regions of *TP53* that are recurrently altered in this subset of patients, akin to patients with *PIK3CA*-mutant tumors. 

The patterns of mutations in *TP53* with *PIK3CA* allowed us to identify four molecular clusters in both PR− and PR+ Luminal breast cancers, namely *PIK3CA*/*TP53*-mutant (Cluster 1), *PIK3CA*-mutant/*TP53* wild-type (Cluster 2), *PIK3CA* wild-type/*TP53*-mutant (Cluster 3), and *PIK3CA*/*TP53* wild-type (Cluster 4). Notably, the prognostic distribution of these clusters differed substantially between ER+/PR− and ER+/PR+ breast cancers. Indeed, while in PR+ Luminal tumors Clusters 2 and 4 were related to better survival, with overlapping curves, in PR− Luminal tumors Cluster 2 followed into in an intermediate risk category for the first 16 years of follow-up, becoming worse after that time. All these diverse correlations highlight the importance of *PIK3CA* and *TP53* analysis in PR− Luminal breast cancer prognostication.

## 4. Materials and Methods

### 4.1. Case Selection and Definitions

We used the CGDS R package to interrogate the Cancer Genomics Data Server [39,40] and download mutational and clinical data related to three breast cancer projects hosted at the Memorial-Sloan-Kettering Cancer Center: the METABRIC project [41], containing 2509 breast cancers samples; the MSK project [42] containing 1918 samples; and the TCGA project, containing 1105 samples. Each sample has both somatic mutational profiles for selected genes, and clinical information. In particular, the TCGA project contains mutational profiles for 20,461 genes, the METABTIC project contains mutational profiles for 173 genes and the MSK project for 474 genes. Moreover, we used gplots and ggplot2 packages [43,44] to perform the clustering analysis and visualize the data. We collected all somatic mutations related to the projects and integrated them to the clinical information and the treatment outcomes. Moreover, we selected all the estrogen receptor positive (ER+) samples reducing our dataset to 3589 samples, and a total of 53,585 somatic mutations in 13,402 genes. 

### 4.2. Statistical Analysis

Comparisons between groups were generally performed using the Student’s *t*-test and test of Equal or Given Proportions. Event-free survival was expressed as the number of months from diagnosis to the occurrence of distant or local relapse or death (disease-related death). Cumulative survival probabilities were calculated using the Kaplan–Meier method. Differences between survival rates were tested with the log-rank test (SPSS version 20.0; IBM). Survival data were censored at five years. A *p* < 0.05 was considered statistically significant. Survival analysis and figures were developed using the R survival and survminer packages [45], and the Kaplan–Meier non-parametric statistic [46]. 

## 5. Conclusions

We demonstrated that ER+/PR− breast cancers are biologically characterized by relevant molecular characteristics in terms of prognostic and predictive information, which could be integrated into the clinical setting to realize the potentials of precision medicine in these clinically, and pathologically, challenging neoplasms. 

## Figures and Tables

**Table 1:** Number ER+ breast cancer samples, according to the PR status from the TCGA, MSK, and METABRIC projects. PR, progesterone receptor.

	TCGA (%)	MSK (%)	METABRIC (%)
PR− (*n* = 959)	110 (12)	396 (41)	453 (47)
PR+ (*n* = 2611)	608 (23)	1031 (40)	972 (37)
Total (*n* = 3570)	718 (20)	1427 (40)	1425 (40)

**Table 2:** The 37 recurrent mutations showing significant differences between ER+/PR− and ER+/PR+ breast cancers according to the test of Equal or Given Proportions.

Mutation	PR+ (%)	PR− (%)	*p* Value
ARID1A_Q766SfsX67	0	2 (0.20)	0.019
ATR_A14S	0	2 (0.20)	0.019
BCL6_K474EfsX26	0	2 (0.20)	0.019
BRAF_V600E	0	2 (0.20)	0.019
CARD11_D200E	0	2 (0.20)	0.019
CDH1_R598X	0	2 (0.20)	0.019
CDH1_E138X	0	2 (0.20)	0.019
CDH1_E497RfsX25	0	2 (0.20)	0.019
AXIN2_S493L	0	3 (0.31)	0.004
GATA3_R364T	0	3 (0.31)	0.005
CDH1_V202CfsX7	0	3 (0.31)	0.006
MUC16_T7149A	0	3 (0.31)	0.007
CCDC82_E175del	0	3 (0.31)	0.008
RUNX1_D123GfsX15	0	4 (0.41)	<0.001
TBX3_W113X	0	4 (0.41)	<0.001
CDH1_T115NfsX53	1 (0.04)	3 (0.31)	0.029
FOXA1_D226N	1 (0.04)	3 (0.31)	0.029
FOXA1_I176M	1 (0.04)	3 (0.31)	0.029
GATA3_X444LfsX63	1 (0.04)	3 (0.31)	0.029
TERT_Promoter	1 (0.04)	3 (0.31)	0.029
TP53_P278S	1 (0.04)	3 (0.31)	0.029
SMAD4_Q245X	1 (0.04)	3 (0.31)	0.029
TP53_I195T	1 (0.04)	5 (0.52)	0.002
ERBB2_E770_A771insGIRD	1 (0.04)	8 (0.83)	0.003
ERBB2_S310F	2 (0.08)	4 (0.41)	0.027
MAP3K1_R364W	2 (0.08)	4 (0.41)	0.027
TP53_H179R	2 (0.08)	4 (0.41)	0.027
TP53_R342X	5 (0.19)	7 (0.72)	0.013
GATA3_D335GfsX17	16 (0.61)	13 (1.35)	0.028
TP53_R175H	21 (0.80)	18 (1.87)	0.006
ESR1_Y537S	29 (1.11)	3 (0.31)	0.024
ESR1_D538G	47 (1.80)	7 (0.72)	0.020
SF3B1_K700E	60 (2.29)	10 (1.04)	0.016
GATA3_X308_splice	70 (2.68)	9 (0.94)	0.002
AKT1_E17K	106 (4.05)	25 (2.60)	0.04
PIK3CA_E545K	251 (9.61)	68 (7.09)	0.019
PIK3CA_H1047R	482 (18.46)	134 (13.97)	0.002

## Supplementary Materials

Supplementary materials can be found at http://www.mdpi.com/1422-0067/20/3/510/s1. Figure S1: Oncoprint visualization of highly recurrent somatic molecular alterations in ER+/PR+ breast cancers (2611 samples). Each row represents a gene, as reported on the right and was sorted by gene alterations frequency (bar plot on the right); types of alterations are color-coded on the basis of the legend on the bottom. Each column represents a sample and was sorted to appreciate the mutual exclusivity across genes. The bar plot on the top represents the number of samples showing alterations in the displayed genes. Cluster analysis, HER2 status, histological type, tumor stage, and menopause status are reported as rows at the bottom of the figure; age at diagnosis is depicted in the top at the bottom. Clustering was performed according to the mutual exclusivity and patterns of mutations. Figure S2: Recurrent somatic alterations in 959 ER+/PR− (A) and in 2611 ER+/PR+ (B) breast cancers (2611 samples). Each row represents an alteration, as reported on the right, each column a sample. Alterations were sorted by frequency, while the samples were sorted to appreciate the mutual exclusivity across alterations. Figures report the 50 most frequent gene alterations. Figure S3: Total number of mutations per samples in ER+/PR− (**A**) and ER+/PR+ (**B**) breast cancer patients. Each bar represents a sample; types of alterations are color-coded on the basis of the legend on the left. Figure S4: Overall survival of ER+/PR− breast cancer patients based on the most frequently altered genes. Survival curves (red, mutant; gray, wild-type) are built according to the Kaplan–Meier method. For each analysis, all samples harboring mutations in one of the other nine genes were excluded. Figure S5: Overall survival of ER+/PR+ breast cancer patients based on the most frequently altered genes. For each analysis, all samples harboring mutations in one of the other nine genes were excluded. Survival curves (red, mutant; gray, wild-type) are built according to the Kaplan–Meier method. Figure S6: Overall survival of ER+/PR− breast cancer patients based on the most frequently altered regions in the *PIK3CA* gene. Survival curves are built according to the Kaplan–Meier method. Figure S7: Overall survival of ER+/PR− breast cancer patients based on the most frequently altered regions in the *TP53* gene. Survival curves are built according to the Kaplan–Meier method. Table S1: Mutations of the tumors included in the analysis.

## References

[1] Thakkar J.P., Mehta D.G. (2011). A review of an unfavorable subset of breast cancer: Estrogen receptor positive progesterone receptor negative. Oncologist.

[2] Arpino G., Weiss H., Lee A.V., Schiff R., De Placido S., Osborne C.K., Elledge R.M. (2005). Estrogen receptor-positive, progesterone receptor-negative breast cancer: Association with growth factor receptor expression and tamoxifen resistance. J. Natl. Cancer Inst..

[3] (2012). WHO Classification of Tumours of the Breast.

[4] Purdie C.A., Quinlan P., Jordan L.B., Ashfield A., Ogston S., Dewar J.A., Thompson A.M. (2014). Progesterone receptor expression is an independent prognostic variable in early breast cancer: A population-based study. Br. J. Cancer.

[5] Van Mackelenbergh M.T., Denkert C., Nekljudova V., Karn T., Schem C., Marme F., Stickeler E., Jackisch C., Hanusch C., Huober J. (2018). Outcome after neoadjuvant chemotherapy in estrogen receptor-positive and progesterone receptor-negative breast cancer patients: A pooled analysis of individual patient data from ten prospectively randomized controlled neoadjuvant trials. Breast Cancer Res. Treat..

[6] Yu K.D., Liu G.Y., Di G.H., Wu J., Lu J.S., Shen K.W., Shen Z.Z., Shao Z.M. (2007). Progesterone receptor status provides predictive value for adjuvant endocrine therapy in older estrogen receptor-positive breast cancer patients. Breast.

[7] Neven P., Pochet N., Drijkoningen M., Amant F., De Smet F., Paridaens R., Christiaens M.R., Vergote I. (2006). Progesterone receptor in estrogen receptor-positive breast cancer: The association between her-2 and lymph node involvement is age related. J. Clin. Oncol..

[8] Viale G., Regan M.M., Maiorano E., Mastropasqua M.G., Dell’Orto P., Rasmussen B.B., Raffoul J., Neven P., Orosz Z., Braye S. (2007). Prognostic and predictive value of centrally reviewed expression of estrogen and progesterone receptors in a randomized trial comparing letrozole and tamoxifen adjuvant therapy for postmenopausal early breast cancer: Big 1-98. J. Clin. Oncol..

[9] Olivotto I.A., Truong P.T., Speers C.H., Bernstein V., Allan S.J., Kelly S.J., Lesperance M.L. (2004). Time to stop progesterone receptor testing in breast cancer management. J. Clin. Oncol..

[10] Burstein H.J., Prestrud A.A., Seidenfeld J., Anderson H., Buchholz T.A., Davidson N.E., Gelmon K.E., Giordano S.H., Hudis C.A., Malin J. (2010). American society of clinical oncology clinical practice guideline: Update on adjuvant endocrine therapy for women with hormone receptor-positive breast cancer. J. Clin. Oncol..

[11] Osborne C.K., Shou J., Massarweh S., Schiff R. (2005). Crosstalk between estrogen receptor and growth factor receptor pathways as a cause for endocrine therapy resistance in breast cancer. Clin. Cancer Res..

[12] Cui X., Schiff R., Arpino G., Osborne C.K., Lee A.V. (2005). Biology of progesterone receptor loss in breast cancer and its implications for endocrine therapy. J. Clin. Oncol..

[13] Creighton C.J., Kent Osborne C., van de Vijver M.J., Foekens J.A., Klijn J.G., Horlings H.M., Nuyten D., Wang Y., Zhang Y., Chamness G.C. (2009). Molecular profiles of progesterone receptor loss in human breast tumors. Breast Cancer Res. Treat..

[14] Maggi A. (2011). Liganded and unliganded activation of estrogen receptor and hormone replacement therapies. Biochim. Biophys. Acta.

[15] Li W., Jia M., Qin X., Hu J., Zhang X., Zhou G. (2013). Harmful effect of erbeta on bcrp-mediated drug resistance and cell proliferation in eralpha/PR−negative breast cancer. FEBS J..

[16] Clarke R., Tyson J.J., Dixon J.M. (2015). Endocrine resistance in breast cancer--an overview and update. Mol. Cell Endocrinol..

[17] Johnston S.R., Saccani-Jotti G., Smith I.E., Salter J., Newby J., Coppen M., Ebbs S.R., Dowsett M. (1995). Changes in estrogen receptor, progesterone receptor, and ps2 expression in tamoxifen-resistant human breast cancer. Cancer Res..

[18] Thomas C., Gustafsson J.A. (2015). Progesterone receptor-estrogen receptor crosstalk: A novel insight. Trends Endocrinol. Metab..

[19] Piscuoglio S., Ng C.K., Murray M.P., Guerini-Rocco E., Martelotto L.G., Geyer F.C., Bidard F.C., Berman S., Fusco N., Sakr R.A. (2016). The genomic landscape of male breast cancers. Clin. Cancer Res..

[20] Fusco N., Geyer F.C., De Filippo M.R., Martelotto L.G., Ng C.K., Piscuoglio S., Guerini-Rocco E., Schultheis A.M., Fuhrmann L., Wang L. (2016). Genetic events in the progression of adenoid cystic carcinoma of the breast to high-grade triple-negative breast cancer. Mod. Pathol..

[21] Kim J., Geyer F.C., Martelotto L.G., Ng C.K.Y., Lim R.S., Selenica P., Li A., Pareja F., Fusco N., Edelweiss M. (2018). Mybl1 rearrangements and myb amplification in breast adenoid cystic carcinomas lacking the myb-nfib fusion gene. J. Pathol..

[22] Fusco N., Colombo P.E., Martelotto L.G., De Filippo M.R., Piscuoglio S., Ng C.K., Lim R.S., Jacot W., Vincent-Salomon A., Reis-Filho J.S. (2016). Resolving quandaries: Basaloid adenoid cystic carcinoma or breast cylindroma? The role of massively parallel sequencing. Histopathology.

[23] Marchiò C., De Filippo M.R., Ng C.K., Piscuoglio S., Soslow R.A., Reis-Filho J.S., Weigelt B. (2015). Piking the type and pattern of pi3k pathway mutations in endometrioid endometrial carcinomas. Gynecol. Oncol..

[24] De Mattos-Arruda L., Bidard F.C., Won H.H., Cortes J., Ng C.K., Peg V., Nuciforo P., Jungbluth A.A., Weigelt B., Berger M.F. (2014). Establishing the origin of metastatic deposits in the setting of multiple primary malignancies: The role of massively parallel sequencing. Mol. Oncol..

[25] Ng C.K., Pemberton H.N., Reis-Filho J.S. (2012). Breast cancer intratumor genetic heterogeneity: Causes and implications. Expert. Rev. Anticancer Ther..

[26] Kandoth C., McLellan M.D., Vandin F., Ye K., Niu B., Lu C., Xie M., Zhang Q., McMichael J.F., Wyczalkowski M.A. (2013). Mutational landscape and significance across 12 major cancer types. Nature.

[27] Fusco N., Lopez G., Corti C., Pesenti C., Colapietro P., Ercoli G., Gaudioso G., Faversani A., Gambini D., Michelotti A. (2018). Mismatch repair protein loss as a prognostic and predictive biomarker in breast cancers regardless of microsatellite instability. JNCI Cancer Spectrum.

[28] Ercoli G., Lopez G., Ciapponi C., Corti C., Despini L., Gambini D., Runza L., Blundo C., Sciarra A., Fusco N. (2017). Building up a high-throughput screening platform to assess the heterogeneity of HER2 gene amplification in breast cancers. J. Vis. Exp..

[29] Fusco N., Bosari S. (2016). HER2 aberrations and heterogeneity in cancers of the digestive system: Implications for pathologists and gastroenterologists. World J. Gastroenterol..

[30] Vuong D., Simpson P.T., Green B., Cummings M.C., Lakhani S.R. (2014). Molecular classification of breast cancer. Virchows Arch..

[31] Russnes H.G., Lingjaerde O.C., Borresen-Dale A.L., Caldas C. (2017). Breast cancer molecular stratification: From intrinsic subtypes to integrative clusters. Am. J. Pathol..

[32] Chakrabarty A., Bhola N.E., Sutton C., Ghosh R., Kuba M.G., Dave B., Chang J.C., Arteaga C.L. (2013). Trastuzumab-resistant cells rely on a her2-pi3k-foxo-survivin axis and are sensitive to pi3k inhibitors. Cancer Res..

[33] Garbe C., Abusaif S., Eigentler T.K. (2014). Vemurafenib. Recent Results Cancer Res..

[34] Fumagalli C., Bianchi F., Raviele P.R., Vacirca D., Bertalot G., Rampinelli C., Lazzeroni M., Bonanni B., Veronesi G., Fusco N. (2017). Circulating and tissue biomarkers in early-stage non-small cell lung cancer. Ecancermedicalscience.

[35] Fda Approved Drug: Neratinib. https://www.accessdata.fda.gov/drugsatfda_docs/label/2017/208051s000lbl.pdf.

[36] Miettinen M., McCue P.A., Sarlomo-Rikala M., Rys J., Czapiewski P., Wazny K., Langfort R., Waloszczyk P., Biernat W., Lasota J. (2014). Gata3: A multispecific but potentially useful marker in surgical pathology: A systematic analysis of 2500 epithelial and nonepithelial tumors. Am. J. Surg. Pathol..

[37] Ng C.K., Schultheis A.M., Bidard F.C., Weigelt B., Reis-Filho J.S. (2015). Breast cancer genomics from microarrays to massively parallel sequencing: Paradigms and new insights. J. Natl. Cancer Inst..

[38] Silwal-Pandit L., Vollan H.K., Chin S.F., Rueda O.M., McKinney S., Osako T., Quigley D.A., Kristensen V.N., Aparicio S., Borresen-Dale A.L. (2014). Tp53 mutation spectrum in breast cancer is subtype specific and has distinct prognostic relevance. Clin. Cancer Res..

[39] Cran—Package Cgdsr. https://cran.r-project.org/web/packages/cgdsr/index.html.

[40] R: The R Project for Statistical Computing. https://www.r-project.org/.

[41] Pereira B., Chin S.F., Rueda O.M., Vollan H.K., Provenzano E., Bardwell H.A., Pugh M., Jones L., Russell R., Sammut S.J. (2016). The somatic mutation profiles of 2,433 breast cancers refines their genomic and transcriptomic landscapes. Nat. Commun..

[42] Razavi P., Chang M.T., Xu G., Bandlamudi C., Ross D.S., Vasan N., Cai Y., Bielski C.M., Donoghue M.T.A., Jonsson P. (2018). The genomic landscape of endocrine-resistant advanced breast cancers. Cancer Cell.

[43] Cran—Package Gplots. https://cran.r-project.org/web/packages/gplots/index.html.

[44] Wickham H. (2018). Ggplot2—Elegant Graphics for Data Analysis.

[45] Cran—Package Survival. https://cran.r-project.org/web/packages/survival/index.html.

[46] Kaplan E.L., Meier P. (1958). Nonparametric estimation from incomplete observations. J. Am. Stat. Assoc..

