# Supplementary figures and images for: Molecular Insights into the Classification of Luminal Breast Cancers: The Genomic Heterogeneity of Progesterone-Negative Tumors
Source: Int J Mol Sci. 2019 Jan 25;20(3):510. doi: 10.3390/ijms20030510 (PMC6386970; doi:10.3390/ijms20030510)

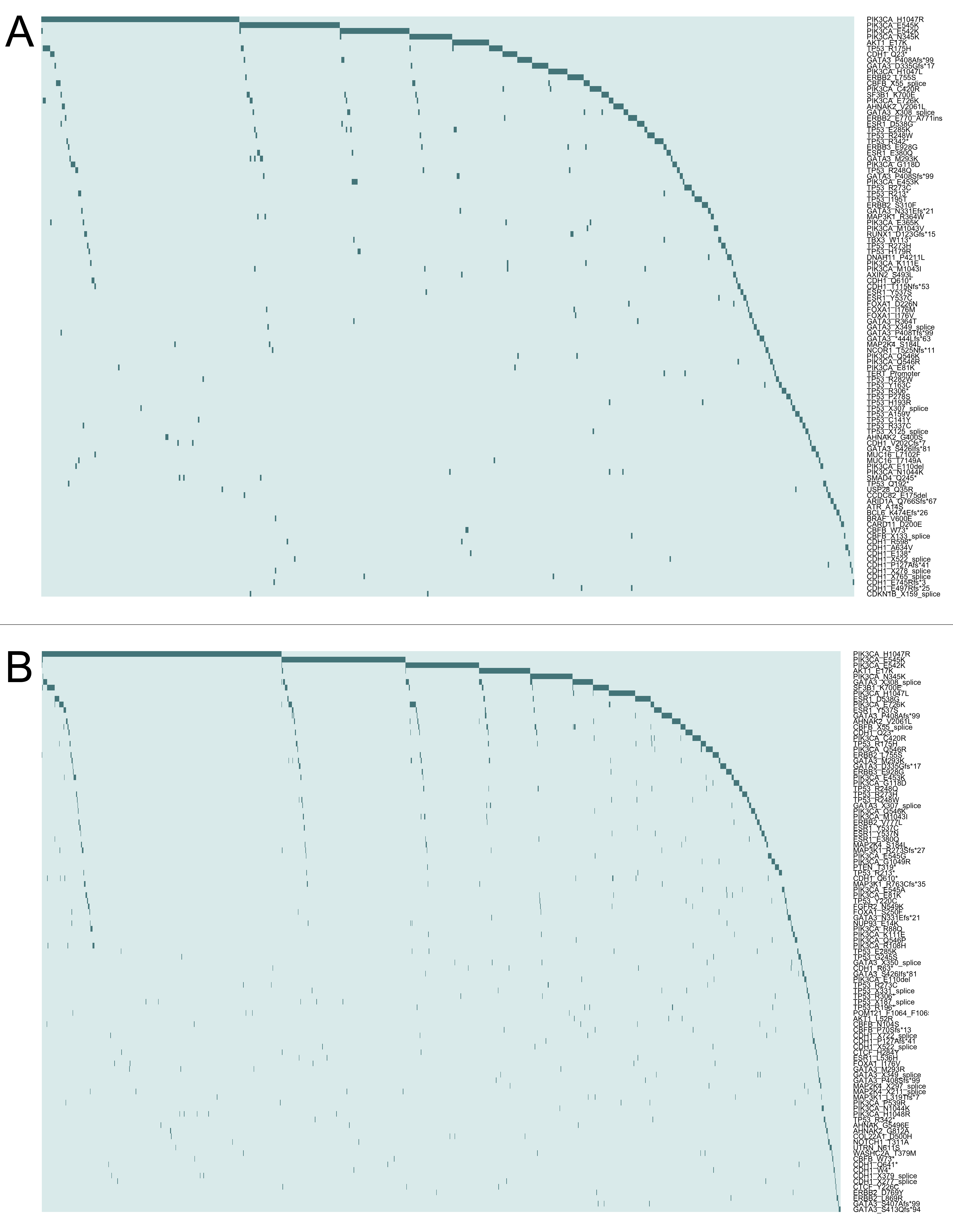

Supplement: Supplementary file 1 [file ijms-20-00510-s001.zip › Lopez et al - IJMS - Supplementary Figure S2.tif]

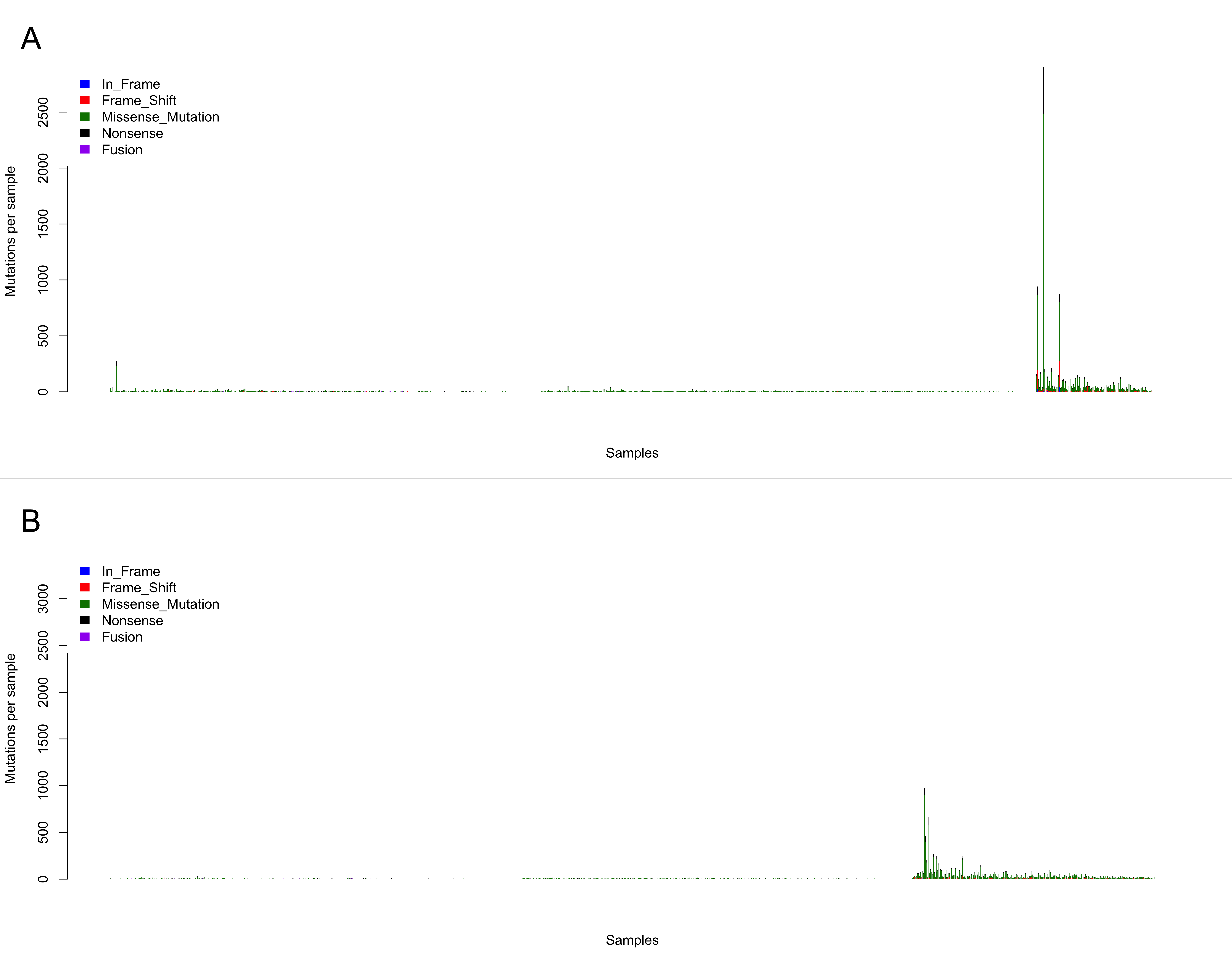

Supplement: Supplementary file 1 [file ijms-20-00510-s001.zip › Lopez et al - IJMS - Supplementary Figure S3.tif]

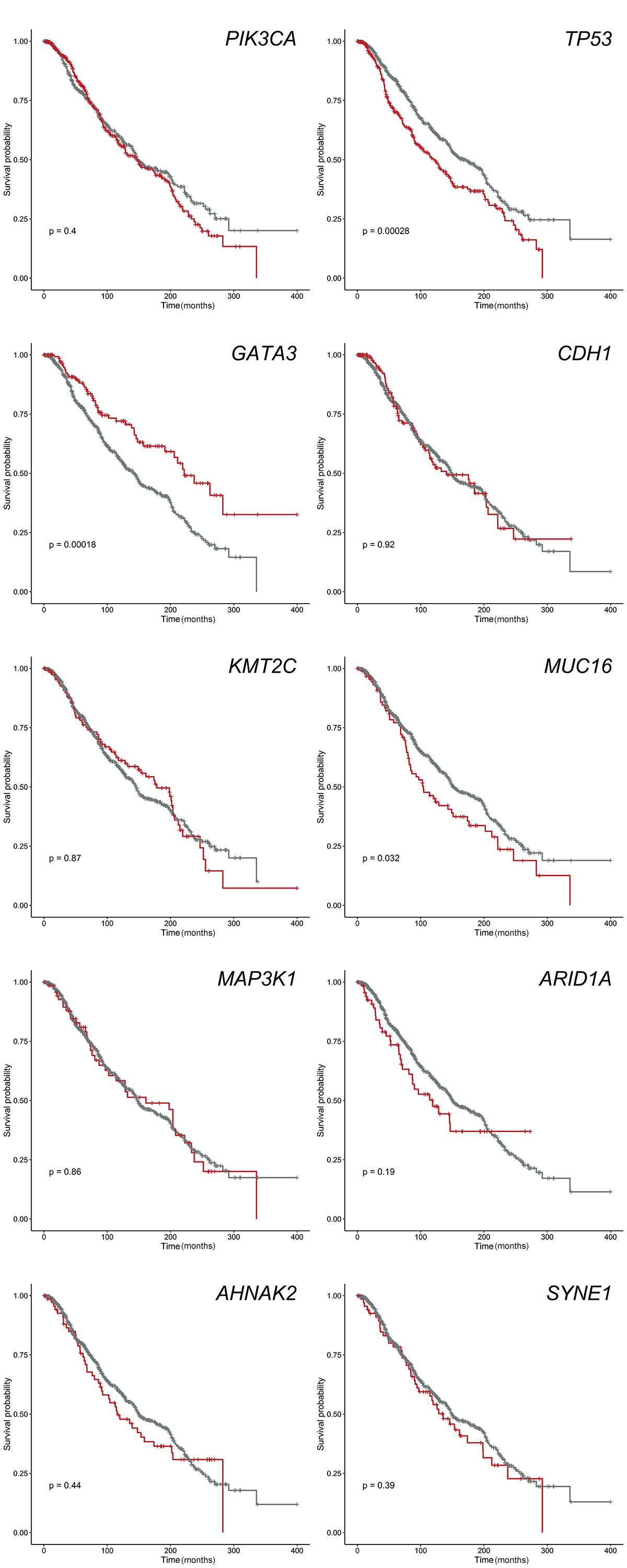

Supplement: Supplementary file 1 [file ijms-20-00510-s001.zip › Lopez et al - IJMS - Supplementary Figure S4 R1.tif]

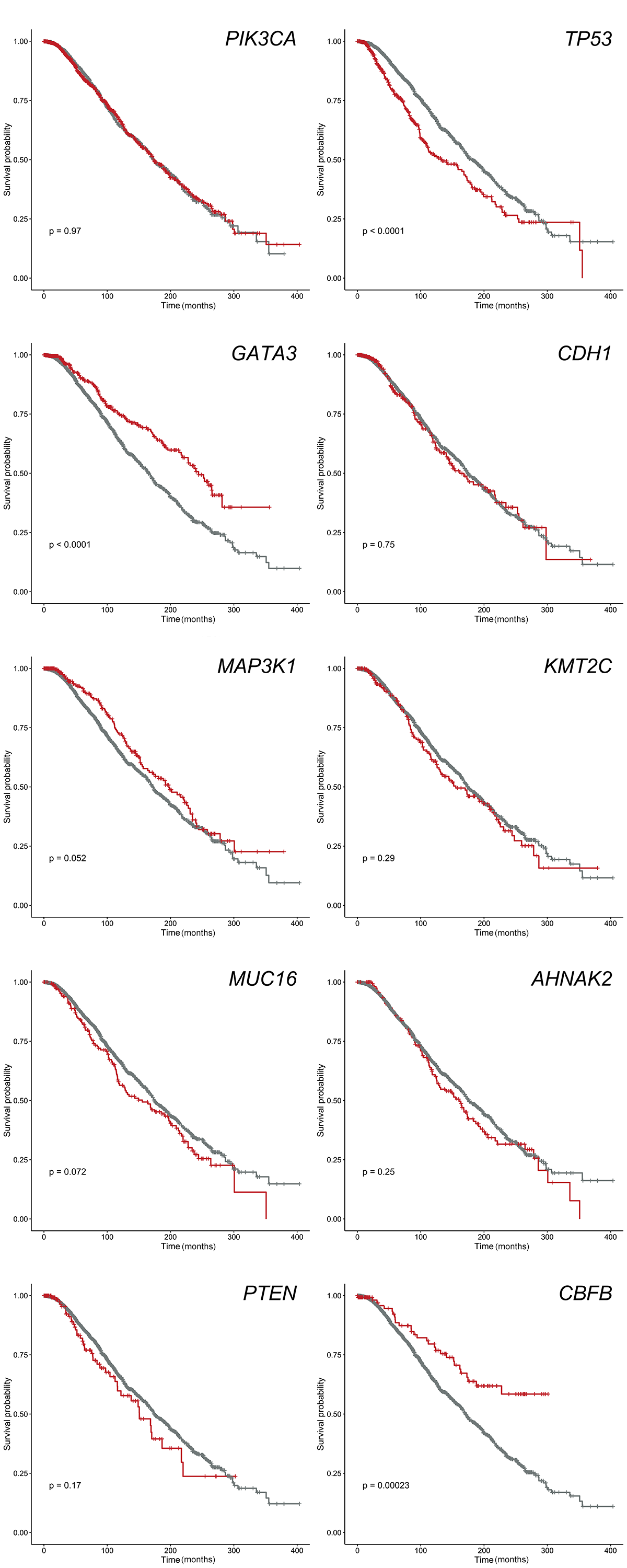

Supplement: Supplementary file 1 [file ijms-20-00510-s001.zip › Lopez et al - IJMS - Supplementary Figure S5 R1.tif]
